# Ten-year results of concentrated autologous bone marrow aspirate transplantation for osteonecrosis of the femoral head: a retrospective study

**DOI:** 10.1186/s12891-019-2797-4

**Published:** 2019-09-05

**Authors:** Yohei Tomaru, Tomokazu Yoshioka, Hisashi Sugaya, Hiroshi Kumagai, Kojiro Hyodo, Katsuya Aoto, Hiroshi Wada, Hiroshi Akaogi, Masashi Yamazaki, Hajime Mishima

**Affiliations:** 1 Department of Orthopaedic Surgery, Faculty of Medicine, University of Tsukuba, 1-1-1 Tennodai, Tsukuba, Ibaraki 305-8575 Japan; 2 Division of Regenerative Medicine for Musculoskeletal System, Department of Orthopaedic Surgery, Faculty of Medicine, University of Tsukuba, 1-1-1 Tennodai, Tsukuba, Ibaraki 305-8575 Japan

**Keywords:** Bone marrow aspirate concentrate, Hip preserving surgery, Osteonecrosis of the femoral head

## Abstract

**Background:**

Idiopathic osteonecrosis of the femoral head (ONFH) occurs at a relatively younger age. It is therefore important to prevent the resultant femoral head collapse and requirement of total hip arthroplasty in these patients. In 2003, we initiated concentrated autologous bone marrow aspirate transplantation (CABMAT), a joint-preserving treatment for ONFH, at our institution. Here, we report the long-term results of CABMAT treatment.

**Methods:**

We retrospectively collated and analyzed the demographic and treatment data of 69 patients (109 hips) with idiopathic ONFH treated with CABMAT between April 2003 and April 2008.

**Results:**

Totally, 44 patients (21 men, 23 women, 80 hips) completed the 10-year follow-up. The follow-up rate was 73.4%, and the mean follow-up period was 12.0 (range, 10.0–15.4) years. The mean age of the patients was 42.2 (range, 16.3–70.5) years. Using the Association Research Circulation Osseous (ARCO) classification system for preoperative analysis, 12, 31, 32, and 5 hips were classified as stages 1, 2, 3, and 4, respectively. The overall rate of conversion to total hip arthroplasty (THA) was 34% (27/80 hips). In a multivariate regression analysis, the preoperative stage of ONFH and the body mass index were found to correlate significantly with conversion to THA. Totally, 43 hips (of 80) were classified as belonging to the pre-collapse stage (i.e., stages 1 or 2). The overall collapse rate and the THA-conversion rate of these hips were estimated to be 49% (21/43) and 14% (6/43), respectively.

**Conclusions:**

On the basis of our long-term findings, the minimally invasive and feasible CABMAT therapy can be utilized as one of a joint-preserving treatment for ONFH.

## Background

Idiopathic osteonecrosis of the femoral head (ONFH) is a refractory hip disorder, which leads to femoral head collapse and hip osteoarthritis. ONFH is defined as an aseptic, ischemic, and atraumatic necrosis of the femoral head and is mainly associated with corticosteroid use and alcohol consumption in Japan [1]. The annual incidence of ONFH in Japan is estimated to be 2.58 cases per 100,000 (range, 1.54–3.66) person-years [2]. The average age at presentation is 50.4 years; ONFH thus involves relatively young and active patients [3]. Total hip arthroplasty (THA) is widely performed in patients with femoral head collapse. However, considering the possibilities of both implant dislocation and expiration, it is desirable to avoid THA at a young age. In particular, compared to osteoarthritis, osteonecrosis is reportedly a high-risk disease, adversely affecting the long-term durability of THA [4]. To avert the requirement for THA at a young age, it is important to prevent femoral head collapse or to minimize its extent. Prevention of both femoral collapse and conversion to THA are the main purposes of joint-preserving treatments, which include femoral and innominate osteotomy, mesenchymal stem cell (MSC) transplantation, and growth factor administration. Several previous studies have also reported the efficacy of bone marrow transplantation in ONFH [5–7].

We initiated concentrated autologous bone marrow aspirate transplantation (CABMAT) for the treatment of ONFH in 2003 [8]. As a part of the transplant procedure, bone marrow is aspirated from the iliac crest of the affected patient and centrifuged twice to separate the buffy coat. The femoral necrotic area is drilled into, and the extracted buffy coat transplanted at the site [8]. The buffy coat extract contains bone marrow cells, MSCs, osteoprogenitor cells, and growth factors [9]. In 2017, we reported the results of CABMAT after a mean follow-up period of 5 years in patients with asymptomatic ONFH [10, 11]. This study aimed to report the results of management of ONFH with CABMAT for > 10 years.

## Methods

In this retrospective study, we followed-up patients treated with CABMAT for ONFH over a minimum 10-year period to evaluate its long-term outcomes. The study was conducted at the University of Tsukuba, Japan, under the supervision of Professor Dr. Masashi Yamazaki and Associate professor Dr. Hajime Mishima.

### Patients

We retrospectively analyzed data of 69 patients (109 hips) diagnosed with idiopathic ONFH, treated using CABMAT, between April 2003 and April 2008. The basic indication for CABMAT was ONFH at the pre-collapse or collapse stage (with the affected portion < 3 mm in diameter), i.e., stage 1 to stage 3A of the Association Research Circulation Osseous (ARCO) classification [12]. Although there were no clinical and radiological data on CABMAT for stages 3B, 3C, and 4 available in the initial period of the study, CABMAT was also performed for patients affected by these stages of ONFH. No patients were treated using the core decompression procedure only.

### ONFH diagnosis and classification

We used the ARCO classification system for the diagnosis, analysis, and classification of ONFH [12]. According to this classification, ONFH is classified into stages 1, 2, 3, and 4 according to the X-ray, computed tomography, scintiscan, and magnetic resonance imaging (MRI) findings. In stage 0, no abnormal findings are seen on any of the imaging studies. In stage 1, abnormal findings are seen only on scintiscan and/or MRI. In stage 2, radiographic abnormalities (e.g. sclerosis, osteolysis, and focal porosis) without crescent sign and collapse are seen. In stage 3, radiographic abnormalities (crescent sign and/or collapse) without progression to osteoarthritis are observed. In stage 4, progression to osteoarthritis is seen.

### Bone marrow aspiration, concentration, and transplantation technique

Several methods of bone marrow transplantation for ONFH exist. Generally, the approaches can be classified as concentration and culture methods. The culture method can deliver a larger number of MSCs compared to the concentration method. However, safety, quality of MSCs, and cost are significant issues [13]. Furthermore, the concentration method can deliver not only MSCs, but also ﻿cytokines and growth factors. For these reasons, the concentration method was adopted at our institution and the novel *CABMAT* approach was developed. CABMAT was performed according to our established protocol, as reported in an earlier study [8]. CABMAT is a single-step, minimally invasive surgical procedure as compared with either osteotomy or THA and does not require a special device, such as a cell sorter.

The marrow was aspirated from both iliac crests of each patient, using a bone marrow harvesting needle (Medical Device Technologies, Inc., Gainesville, FL, USA) and transferred into a bone marrow collection bag. After centrifugation at 1200 g for 10 min (Centrifuge Kubota 9800, Kubota, Japan), erythrocytes were transferred from the main bag to a satellite bag. After a second round of centrifugation (3870 g for 7 min), the plasma was transferred from the main bag to another satellite bag, which left the buffy coat layer in the main bag.

During transplantation, we first performed core decompression of the femoral head using a trephine with a thickness of 4.8 mm (Iso Medical Systems, Tokyo, Japan). The drill was inserted percutaneously into the center of the necrotic area. After core decompression, drilling was performed multiple times using a 2.4-mm diameter Kirschner wire. To perforate the interface between the normal bone and necrotic bone, the surgeon drilled repeatedly at sites anteromedial and posterolateral to the core decompression tract. We then used a monitor with biplane fluoroscopic guidance to transplant the buffy coat at the drilled sites.

### Colony-forming-unit-fibroblast (CFU-F) assay

We performed the CFU-F assay to evaluate the presence of MSCs in the buffy coat. The analysis was also performed according to our previously published method [8]. After 2 weeks of culture at 37 °C in a humidified atmosphere of 5% carbon dioxide, the number of colonies > 2 mm in diameter were counted. The number of MSCs was expressed as the number of CFU-Fs per 10^6^ nucleated cells. Since the CFU-F assay had not been performed during the initial period of the study, we could only collect these data for 53 of 80 hips (66%). As this was a significant loss, CFU-F assay data were not included in the statistical analyses.

### Outcome measures

The rate of conversion of the treatment protocol from CABMAT to THA was set as the primary endpoint of this study. The indication for THA in this study was the development of stage 4, painful ONFH. The percentage of hips developing femoral head collapse and factors predictive of conversion to THA were set as secondary endpoints. Treatment efficacy was estimated using the rate of conversion to THA, which was indicative of the effect of CABMAT on the natural history of ONFH. All hips were accordingly classified into THA-conversion or THA non-conversion groups for the univariable analysis. Univariable and multivariable analyses were also performed to evaluate factors predictive of THA conversion. The mean age, body mass index (BMI), and follow-up period were compared between both groups using the unpaired *t* test. The chi-square test was used to compare the sex distribution of the patients between both groups. We compared etiologies and stages of ONFH (as per the ARCO classification) between the two groups using residual analysis. THA was set as the objective variable for the multivariable logistic analysis. Factors including age, sex, etiology, BMI, follow-up period, and preoperative stage were set as the explanatory variables. A *p*-value < 0.05 was considered statistically significant. All statistical analyses were performed with SPSS Statistics version 15 (IBM, Armonk, NY) software.

### Radiographic evaluation of femoral head collapse

Anteroposterior and lateral radiography of the treated hip was performed at regular intervals during the patients’ follow-up hospital visits. The presence of femoral head collapse was determined using circular template overlays [14] on anteroposterior or lateral radiographs, irrespective of the site of the collapse. All radiographs were independently evaluated by two specialist orthopedic surgeons.

## Results

**Fig. 1:**
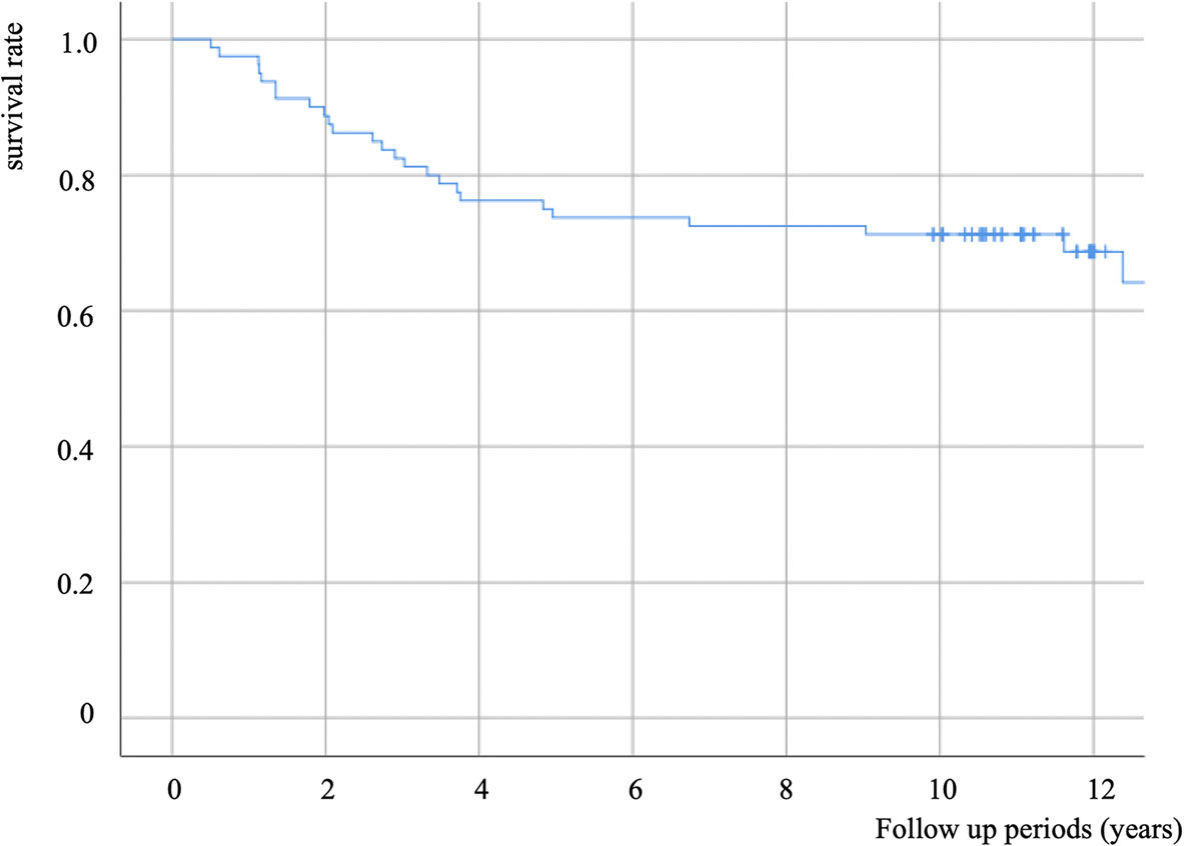
Survival curve (end point: conversion to total hip arthroplasty)

We were able to conduct follow-up evaluations in 44 of the 69 patients (21 men, 23 women; 80 of 109 hips) for > 10 years. The follow-up rate was 73.4% (80/109). The mean age of the patients was 42.2 (range, 16.3–70.5) years, and the mean follow-up period was 12.0 (10.0–15.4) years. The mean CFU-F count per 10^6^ nucleated cells was 2.33 ± 2.67. The overall THA-conversion rate was 34% (27/80). The mean period before conversion to THA was 4.4 (range, 0.5–15; median, 2.9) years. The survival curves are shown in Fig. 1. The rate of conversion to THA was 25% (3/12), 10% (3/31), 50% (16/32), and 100% (5/5) for ONFH stages 1, 2, 3, and 4, respectively. THA conversion rate was 14% (6/43) for pre-collapsed ONFH stages (i.e., stages 1 or 2). Characteristics of the THA-conversion and non-conversion groups are shown in Table 1. Factors including BMI and rate of occurrence of ONFH stages 3B and 4 were significantly higher in the THA-conversion group. The preoperative stage of osteonecrosis and the patient’s BMI were found to be significantly correlated with conversion to THA in the multivariable statistical analysis. Metabolic syndrome (diabetes mellitus, hypertension, and hyperlipidemia) was present in 40% (21/53) and 44% (12/27) of the THA non-converted and THA converted groups, respectively, with no significant difference between them (chi-square test, *p* = 0.67).

**Table 1:** Comparison of characteristics of the THA-conversion and THA non-conversion groups

	THA^b^-conversion group	THA^b^ non-conversion group	*P*-value	Statistical method
n	27	53	–	–
Men/Women	11/16	30/23	n.s.	χ^2^ test
Age	43.7(21.5–70.5)	41(16–71)	n.s.	*t*-test
BMI	24 (20–32)^a^	21.9 (17–30)	*p* < 0.01	*t*-test
Presence of metabolic syndrome	40% (21/53)	44% (12/27)	n.s.	χ^2^ test
Follow up period	13 (11–15)	11.8 (10.0–15.0)	n.s.	*t*-test
Etiology				
Steroid	19 (70%)	37 (70%)	n.s.	Residual analysis
Alcohol	5 (19%)	14 (26%)		
Idiopathic	3 (11%)	2 (4%)		
Preoperative stage				
1	3	9	n.s.	
2	3	28^a^	*p* < 0.01	
3	16	16	n.s.	
4	5^a^	0	*p* < 0.01	

^a^Statistically significant values, ^b^THA (total hip arthroplasty)

Of the 80 hips, 43 were classified as belonging to the pre-collapse stage (i.e., stages 1 or 2). The overall collapse rate for these hips was 49% (21/43). The collapse rates of hips belonging to ONFH stages 1 and 2 were 50% (6/12) and 48% (15/31), respectively. THA conversion rate in pre-collapse stage (i.e., stages 1 or 2) was 14% (6/43), respectively. There were no cases of tumorigenic transformation or infection following CABMAT.

## Discussion

We studied the long-term outcomes of CABMAT. In summary, the overall conversion to THA rate was 34% (27/80 hips). Preoperative stage and BMI were found to correlate significantly with conversion to THA in the multivariate regression analysis. Forty-three hips (of 80) were classified as belonging to the pre-collapse stage (i.e., stages 1 or 2). The overall collapse and THA-conversion rates of these hips were 49% (21/43) and 14% (6/43), respectively.

The exact pathophysiology of ONFH is still unclear. Studies have suggested genetic predisposition, metabolic factors, mechanical stress, vascular failure, and raised intraosseous pressure as possible causes [15]. Intake of corticosteroids and excessive alcohol not only induce development of the fat-storing phenotype among the bone-marrow progenitor cells, which leads to an insufficient supply of normal cells [6], but also cause fat embolisms and arteriosclerosis, which result in a reduced blood supply to necrotic areas [16]. Corticosteroids reportedly have an adverse effect on bone renewal by decreasing the number of progenitor cells [17]. Kuroda et al. reported good clinical outcomes of local administration of gelatin hydrogel impregnated with recombinant human fibroblast growth factor (FGF)-2 for the treatment of the pre-collapse stage of ONFH [18]. This suggests that FGF pathology plays a role in necrosis, and its replacement with functional growth factor will prevent femoral head collapse. Considering that osteonecrosis may be caused by vascular failure, FGF failure, raised intraosseous pressure, and an insufficiency of progenitor cells at the necrotic sites, we used CABMAT as a therapeutic modality. In CABMAT, the isolated buffy coat, which contains MSCs and various growth factors (e.g., platelet-derived growth factors, FGFs, and transforming growth factor-β), is transplanted to the necrotic area after core decompression [9]. Core decompression effectively lowers the intraosseous pressure within the necrotic area, thereby inducing migration of MSCs and various growth factors from healthy bone into the necrotic tissue [19].

The collapse and THA-conversion rates in the pre-collapse stages were found to be 49% (21/43) and 14% (6/43), respectively. The overall THA-conversion rate was 34% (27/80). There are several reports on the natural history and progression of ONFH. The rate of conversion to THA in our present study was lower than that reported previously [20], while no significant difference was observed as compared to that reported by others [21, 22] (Table 2).

**Table 2:** Comparison of our study results with the natural history of ONFH and outcomes of other treatments

	Treatment	Number of hips	Follow-up period (years)	Collapse Rate (%)	THA -conversion rate (%)
this study	CD^a^ + BMC^b^	80	12	60% (20/33)	34% (27/80)
Hernigou P 2006 [21]	natural history	121	14	77% (93/121)	75% (91/121)
Nam KW 2008 [22]		105	8.6	59% (62/105)	44% (46/105)
Koo KH 1995 [20]		19	2	79% (15/19)	68% (13/19)
Learmonth ID 1990 [23]	CD^a^	41	2.6	83% (34/41)	–
Gangji V 2011 [7]		11	5	73% (8/11)	27% (3/11)
Koo KH 1995 [20]		18	2	72% (13/18)	72% (13/18)
Hernigou P 2009 [6]	CD^a^ + BMC^b^	534	13	30% (160/534)	18% (96/534)
Hernigou P 2002 [5]		189	7	–	18% (34/189)
Gangji V 2011 [7]		13	5	23% (3/13)	–
Kuroda Y 2016 [18]	gelatin hydrogel + FGF^c^	10	1	10% (1/10)	0% (0/10)
Lieberman JR 2004 [24]	CD^b^ + fibula allograft + BMP^d^	17	4.4	18% (3/17)	18% (3/17)
Mont A 1996 [25]	osteotomy	77	11.5	–	24%
Sugioka Y 1978 [26]		41	2.5	6% (stage 1, 2)	–

(ONFH-osteonecrosis of the femoral head, ^a^*CD* Core decompression, ^b^*BMC* Bone marrow concentrate, ^c^ *FGF* Fibroblast growth factor, ^d^*BMP* Bone morphogenetic protein)

Hernigou et al. reported a THA-conversion rate of 18% in ~ 13 years [6], which was lower than the rate observed in this study. By definition, the protocol of Hernigou et al. only included radiographic stages 1 and 2 [6]. In the present study, the THA-conversion rate in the pre-collapse stages (1 and 2) was 14% (6/43), which was somewhat lower than that reported by Hernigou et al. [6]. Hernigou et al. had used a cell separator for the separation of MSCs, while in our study, we used an ordinary blood bag to separate the buffy coat after simple centrifugation. Using this method, we achieved a mean CFU-F count of 2.33 ± 2.67 per 10^6^ nucleated cells, while Hernigou et al. reported a much higher mean CFU-F count of 12.4 ± 3.4 per 10^6^ nucleated cells [5]. Despite the difference in CFU-F count, in the pre-collapsed stages, the THA conversion rate did not significantly differ between our study and Hernigou et al’s.

Several studies on regenerative therapies (other than bone marrow transplantation) for ONFH have reported lower THA-conversion rates than that observed in our present study [18, 24]. There are also several published studies on bone marrow transplantation. Though positive outcomes of bone marrow transplantation have been reported in systematic reviews, there are no randomized control trials providing level 1 evidence for this therapy [27, 28].

Although, our study showed a better outcome with CABMAT than that observed with the natural progression of ONFH, the overall result is not satisfactory. Cultivation of MSCs, combined transplantation with platelet-rich plasma, filling the core decompression tract with artificial bone for mechanical support, and using a gelated buffy coat in combination with FGF are methods from earlier studies that can be adopted to improve the outcomes of CABMAT [6, 18].

Mont et al., Sugioka et al. and Dean et al. reported THA-conversion rates of 24, 6, and 67% over follow-up periods of 11.5, 2.5, and 8.2 years, respectively, for ONFH treated with femoral osteotomy [25, 29, 30]. The THA-conversion rate reported by Sugioka et al. was lower, while the rate reported by Dean et al. was higher than that of our study [30]. However, since the follow-up period and patient backgrounds were different, it is difficult to simply compare the THA conversion rate. Sugioka et al. have suggested that > 36% of the weight-bearing area should be intact to perform an adequate osteotomy [29]. Therefore, ONFH with a broad necrotic area may not benefit from a femoral osteotomy. Furthermore, osteotomy is a highly technically demanding procedure that can result in an unevenness of the femoral weight-bearing surface. In contrast, our treatment approach does not require technical expertise, and it is cost-effective and minimally invasive.

In the comparative univariate statistical analysis between the THA-conversion and THA non-conversion groups, BMI along with rate of occurrence of stage 4 were found to be significantly higher in the THA-conversion group. In the multivariable statistical analysis, both preoperative stage of ONFH and BMI correlated significantly with conversion to THA. Based on these findings, we suggest that an advanced class and stage of ONFH and a high body weight are predominant risk factors for conversion to THA. Mechanical stress due to body weight is considered as one of the factors responsible for femoral head collapse and conversion to THA. According to a report of Kanazawa et al., in Japan, BMI ≥ 25 is defined as obesity [31]. Since the average BMI in the THA-conversion group in this study was 24 kg/m^2^, which is lower than the cut-off value for obesity, the extent of the weight’s contribution to mechanical stress to the femoral head is unknown. It is known that obesity is closely associated with metabolic syndromes such as diabetes mellitus, hypertension, and hyperlipidemia. But presence rate of metabolic syndromes was not significant different between THA conversion group and THA non-conversion group. While the preoperative stage of ONFH is not changeable, body weight is a relatively controllable factor. Regulation of body weight might prevent or delay femoral head collapse and conversion to THA.

There are several limitations to this study. Since this is a retrospective study, having a control group was not possible, which was an important limitation. Secondly, since the etiology, preoperative stages, classification systems, and follow-up periods differed among the study patients, it was difficult to compare the overall effect of CABMAT with the natural progression of ONFH, core decompression, or other treatments.

## Conclusion

Both collapse and THA-conversion rates in the pre-collapse stage of ONFH were estimated at 49% (21/43) and 14% (6/43), respectively. The overall THA-conversion rate of ONFH was 34% (27/80). Based on our findings, the minimally-invasive and feasible CABMAT therapy can be considered as one of the alternatives for joint-preserving treatment of ONFH, especially in stages 1 and 2.

Our group started CABMAT in 2003. By reporting further long-term results of this therapeutic modality and comparing its outcomes with those of other existing treatments, we aim to ultimately determine the best joint-conserving treatment for ONFH.

## Data Availability

The datasets used and/or analyzed during the current study are available from the corresponding author on reasonable request.

## Abbreviations

ARCO: Association Research Circulation Osseous
BMI: Body mass index
CABMAT: Concentrated autologous bone marrow aspirate transplantation
CFU-F: Colony-forming-unit-fibroblast
FGF: Fibroblast growth factor
MRI: Magnetic resonance imaging
MSC: Mesenchymal stem cell
ONFH: Osteonecrosis of the femoral head
THA: Total hip arthroplasty
